# Genome-wide characterization and transcriptional responses of *Tuta absoluta* glutathione S-transferases to chlorantraniliprole and spinetoram

**DOI:** 10.1093/jisesa/ieag073

**Published:** 2026-07-21

**Authors:** Chanling Ma, Bo Xu, Wumuerhan Patima, Guifen Zhang, Cong Huang, Yibo Zhang, Fanghao Wan

**Affiliations:** State Key Laboratory for Biology of Plant Diseases and Insect Pests, Key Laboratory for Prevention and Control of Invasive Alien Species of Ministry of Agriculture and Rural Affairs, Institute of Plant Protection, Chinese Academy of Agricultural Sciences, Beijing, China; College of Agriculture, Xinjiang Agricultural University, Urumqi, China; State Key Laboratory for Biology of Plant Diseases and Insect Pests, Key Laboratory for Prevention and Control of Invasive Alien Species of Ministry of Agriculture and Rural Affairs, Institute of Plant Protection, Chinese Academy of Agricultural Sciences, Beijing, China; College of Agriculture, Xinjiang Agricultural University, Urumqi, China; State Key Laboratory for Biology of Plant Diseases and Insect Pests, Key Laboratory for Prevention and Control of Invasive Alien Species of Ministry of Agriculture and Rural Affairs, Institute of Plant Protection, Chinese Academy of Agricultural Sciences, Beijing, China; State Key Laboratory for Biology of Plant Diseases and Insect Pests, Key Laboratory for Prevention and Control of Invasive Alien Species of Ministry of Agriculture and Rural Affairs, Institute of Plant Protection, Chinese Academy of Agricultural Sciences, Beijing, China; State Key Laboratory for Biology of Plant Diseases and Insect Pests, Key Laboratory for Prevention and Control of Invasive Alien Species of Ministry of Agriculture and Rural Affairs, Institute of Plant Protection, Chinese Academy of Agricultural Sciences, Beijing, China; State Key Laboratory for Biology of Plant Diseases and Insect Pests, Key Laboratory for Prevention and Control of Invasive Alien Species of Ministry of Agriculture and Rural Affairs, Institute of Plant Protection, Chinese Academy of Agricultural Sciences, Beijing, China

**Keywords:** *Tuta absoluta*, glutathione S-transferase, spinetoram, chlorantraniliprole, metabolic resistance

## Abstract

*Tuta absoluta* is a major invasive pest of Solanaceous crops, largely managed through chemical interventions. Despite its economic importance, the genomic landscape of its detoxification machinery remains poorly understood. We performed a genome-wide identification of 23 GST genes (*TabsGSTs*) in *T. absoluta*, characterized by amino acid lengths of 147-289 aa and a predominantly 1- or 5-exon genomic architecture (∼65%). The identification of four tandemly arranged gene clusters highlights potential evolutionary hotspots for insecticide resistance. Phylogenetic analysis categorized these genes into seven subfamilies, with the lineage-specific expansion of the Epsilon class indicating its key role in xenobiotic metabolism. Transcriptional profiling revealed divergent responses to insecticides: chlorantraniliprole exposure (LC_50_) largely failed to induce *TabsGST* expression, whereas spinetoram LC_50_ treatment triggered significant upregulation of *TabsGSTe6* and *TabsGSTu1*. This study elucidates the molecular characteristics and expression dynamics of the GST family in *T. absoluta*, offering vital molecular targets for resistance monitoring and the design of targeted control measures.

## Introduction

The South American tomato leafminer, *Tuta absoluta* (Meyrick) (Lepidoptera: Gelechiidae), is a major invasive pest that inflicts severe economic losses on tomato production globally. Despite the continued reliance on chemical insecticides as the cornerstone of *T. absoluta* management, intensive selection pressure has triggered the widespread evolution of resistance, thereby undermining control efficacy. To date, resistance across various chemical classes—including pyrethroids, abamectin, cartap, organophosphates, indoxacarb, spinosad, and diamides—has been confirmed (Siqueira et al. 2000, Silva et al. 2011, Roditakis et al. 2013, Campos et al. 2015). Even newer compounds with novel mechanisms, such as chlorantraniliprole and spinetoram, are facing increasing resistance in field populations. Given its high fecundity and overlapping generations, the rapid proliferation of resistant genotypes remains a critical threat (Desneux et al. 2011). Consequently, characterizing the underlying resistance mechanisms is imperative to inform and optimize future resistance management frameworks.

Insecticide resistance is fundamentally categorized into target-site, metabolic, and penetration mechanisms. Metabolic resistance, in particular, stems from the sophisticated regulation of detoxification enzyme systems. Glutathione S-transferases (GSTs), as indispensable Phase II enzymes, are ubiquitous across eukaryotes and prokaryotes and serve as cornerstones of metabolic defense (Sheehan et al. 2001). They orchestrate the detoxification of a wide array of xenobiotics—ranging from synthetic pesticides to endogenous toxins—by catalyzing the conjugation of reduced glutathione (GSH) to electrophilic centers (Hayes et al. 2005, Ranson and Hemingway 2005, Berenbaum and Johnson 2015). Beyond their catalytic prowess, GSTs function as ligand-binding proteins (ligandins) for hydrophobic compounds that are not processed enzymatically. This non-catalytic sequestration is crucial for the intracellular transport and homeostasis of diverse molecules, including fatty acids, heme derivatives, and hormones (Hayes and Pulford 1995).

Insect GSTs predominantly comprise 6 cytosolic subfamilies: Delta, Epsilon, Omega, Sigma, Theta, and Zeta (Enayati et al. 2005). Recent genomic advancements have facilitated the comprehensive identification of GSTs across diverse insect taxa. In *Bactrocera dorsalis*, 17 GSTs were identified, some of which were highly expressed in key detoxifying organs—the midgut, fat body, and malpighian tubules—under insecticide stress (Hu et al. 2014). Genomic and transcriptomic analyses of *Leptinotarsa decemlineata* revealed 30 GST genes, with several exhibiting marked induction following treatment with cyhalothrin, fipronil, or endosulfan (Han et al. 2016). Furthermore, in *Plutella xylostella*, 22 cytoplasmic GSTs have been identified, with the Delta and Epsilon subfamilies specifically upregulated in the detoxifying tissues of multiple resistant strains, highlighting their role in conferring insecticide tolerance (You et al. 2015).

As a globally invasive and destructive pest, *T. absoluta* poses a severe threat to solanaceous crop production, largely due to its rapid evolution of insecticide resistance. This study presents a comprehensive genome-wide characterization of the *T. absoluta* glutathione S-transferase (GST) gene family. We identified 23 GST genes, unravelling their genomic architecture through exon-intron analysis and chromosomal mapping. Phylogenetic reconstruction with 6 related taxa further elucidated the evolutionary trajectory of this family. Moreover, we functionally investigated the transcriptomic response of these GSTs to chlorantraniliprole and spinetoram via RT-qPCR. This genomic framework not only offers a foundational resource for identifying key resistance determinants but also enables a more nuanced understanding of detoxification mechanisms. Ultimately, these insights provide a blueprint for advancing next-generation management strategies, including RNAi-based interventions and optimized insecticide resistance management (IRM) programs.

## Materials and Methods

### Insects

The *T. absoluta* strain was established from individuals collected in Beijing, China (116.22°E, 40.10°N) in April 2024. The culture was maintained on tomato plants in a climate-controlled chamber (25 ± 1 °C, 60 ± 5% RH, 16:8 L:D h) without insecticide selection pressure. Adults were supplemented with 10% honey solution provided via cotton wicks suspended within the cages. Fresh tomato seedlings served as oviposition substrates; progeny were collected post-hatching for experimental use.

A susceptible tomato cultivar (*Solanum lycopersicum* cv. ‘Provence’) was used for host maintenance and bioassays. Analytical standard chlorantraniliprole (98.2% purity) and spinetoram (81.63% purity) were sourced from Beijing Qincheng Yixin Science and Technology Development Co., Ltd. and Qilu Pharmaceutical Co., Ltd., respectively.

### Identification of GST Genes in *T. absoluta*

To identify candidate GST genes, 54 GST sequences from FlyBase (https://flybase.org/) were used as queries in a BLASTP analysis (*E*-value < 1e-5) against the *T. absoluta* genome. The resulting candidates were then systematically verified using HMMER v3.1b2 to confirm the presence of characteristic GST domains. This involved searching against the Pfam-A database for specific Hidden Markov Model (HMM) profiles, including various N-terminal (PF02798.20, PF13409.6, PF13417.6, PF17172.4) and C-terminal motifs (PF00043.25, PF13410.6, PF14497.6, PF17171.4).

### Gene Structure Analysis and Chromosomal Mapping

The exon-intron organizations of *T. absoluta* GST genes were characterized by submitting GFF3-derived coordinates to the GSDS 2.0 server. To illustrate their physical positions across the genome, chromosomal distribution maps were generated using a custom Python framework, ChrLocPlotter (https://github.com/jackiexls/ChrLocPlotter, accessed on 10 June 2022).

### Phylogenetic Analysis

The evolutionary history of *T. absoluta* GSTs was inferred by comparing 160 protein sequences across seven insect taxa, including *Bombyx mori*, *Cydia pomonella*, *Tribolium castaneum*, *Nasonia vitripennis*, *Nilaparvata lugens*, and *D. melanogaster* (Xu et al. 2020, Hu et al. 2022, Meng et al. 2022). Amino acid sequences were aligned via MAFFT v7 and automatically trimmed using trimAl v1.2 (Capella-Gutiérrez et al. 2009). Phylogenetic relationships were reconstructed using a maximum-likelihood framework in RAxML (version 8.2.12), with the LG + I substitution model selected as the optimal fit by ProtTest v3.4.2 (Darriba et al. 2011). The final tree was visualized using FigTree v1.4.3 and aesthetically enhanced in Adobe Illustrator CC 2017.

### Spatiotemporal Expression Profiles of *TabsGST* Genes

To characterize the developmental expression patterns of GSTs, total RNA was isolated from *T. absoluta* at various stages (eggs, first-fourth instar larvae, male/female pupae, and male/female adults) using TRIzol reagent. Genomic DNA-free cDNA was synthesized from the purified RNA using Hifair® III 1st Strand cDNA Synthesis SuperMix in a 20 μL reaction volume. The thermal profile for reverse transcription consisted of 25 °C (5 min), 55 °C (15 min), and 85 °C (5 min). Each sample was represented by five independent biological replicates, with two technical replicates per sample to ensure experimental consistency during the subsequent RT-qPCR assays.

A total of 20 *TabsGST* genes were selected to analysis their spatiotemporal expression profiles, gene-specific primers were designed using Primer Premier 5.0 software. and *RPL5* was selected as the reference gene for *T. absoluta* (Camargo et al. 2016). All primers were synthesized by Sangon Biotech (Shanghai, China) Co., Ltd., and their specific sequences are provided in Table 1.

**Table 1.** qPCR primers for GST subfamily genes

Primer name	F	R
RPL5	CAGTCGTCGAGCCAGCAACA	TCCCGCATTGAAGGAGACCA
q*TabsGSTd1*	CCAGCCGACAAGGACAAGA	GGACACAGAGGCGACGAGA
q*TabsGSTd2*	TTGTATCTCCCAATCCTGTT	GCTCTGTTTTCGTCCTTTC
q*TabsGSTe1*	TTTGTCTGTCGTCACTTCAT	ATTGAATCATACTCCGTAACCACTC
q*TabsGSTe2*	GACAGAGTAGAGAGAGCAGCA	CAACCAAACCCTCATAAAA
q*TabsGSTe3*	AAGAGGCTTTGGGATTAGA	AACAGCGGGACAGTATGTA
q*TabsGSTe4*	TTCACAAAGATAAACCCGC	CACGATCAATGGCAAAGAG
q*TabsGSTe5*	GCAAGAGAACAAGATACACCT	TGGAAATAGAATACCGCAC
q*TabsGSTe6*	AAATGATCGACGTCAAGTTA	AGATGCCAGTGTCAAAGAA
q*TabsGSTe7*	CCTCATAAAAGAAAAAGCCA	TTCGTCAATCGGAACAAAT
q*TabsGSTe8*	GATAAAGCAAAGTACCCTATTAC	TGACGACCTTACCCAAATA
q*TabsGSTt1*	CAAGACCCAAAACGAATTG	GTCAGCGACCGTCACAGTA
q*TabsGSTs1*	GAAGAAGGAGGAAGTGAAGA	CACGATACCAATGAGGATG
q*TabsGSTs2*	ATACGAATCCGACGAAGAG	CATACCCGCAAATACAAAG
q*TabsGSTz1*	GGAAGGGCATACCATTTGA	CGGCGTACTTGGAGACAGA
q*TabsGSTz2*	CAGAACCTCGTCGTCCTCA	TCTCGTCCCCAACACAGTA
q*TabsGSTo1*	ACGCCAAGAAGGTCAAATA	CACTACGCTCTCGAACAGG
q*TabsGSTo2*	TCCTGGCTTTGCCTCTCTT	GGCTGGGGTGTTCTTCATT
q*TabsGSTo3*	GTATCCTGAAACCAGTCGC	ACCTCATAGTCCAGCCCTT
q*TabsGSTu1*	AGGGCAATCGTAAACCACC	CTTTCTTCAAGCCAAGGGG
q*TabsGSTu2*	AAGCCACTAAAGGACGACC	CCCATCCAACAGCAAGAAT

### Laboratory Toxicity Bioassays

Toxicity bioassays were conducted in accordance with the IRAC-approved leaf-dip method (https://irac-online.org/methods/tuta-absoluta-larvae/). A 1000 mg/L stock solution was prepared by dissolving 0.01 g of each technical-grade insecticide in 10 mL of methanol. Serial dilutions were then performed using distilled water containing Tween-20 as a surfactant to obtain the test concentrations listed in Table 2. The geometric dilution series was optimized to establish a high concentration yielding 80% to 100% mortality and a low concentration yielding 10% to 20% mortality. Bioassays were considered valid only when control mortality remained below 10% to 15% and did not exceed the mortality observed at the lowest insecticide concentration.

**Table 2.** Concentration gradient settings for chlorantraniliprole and spinetoram

Test agent	Concentration gradient (mg/L)					
Chlorantraniliprole technical grade 98.2% TC	0.04	0.08	0.16	0.32	0.64	1.28
Spinetoram technical grade 81.63% TC	0.05	0.1	0.2	0.4	0.8	1.6

### Transcriptional Response of GSTs to Insecticide Exposure

To investigate the induction of GST genes under insecticide stress, *T. absoluta* individuals were treated with LC_50_ concentrations of chlorantraniliprole (0.216 mg/L) and spinetoram (0.339 mg/L) for 12, 24, 48, and 72 h. Total RNA isolation and subsequent cDNA synthesis were conducted as detailed in “Spatiotemporal Expression Profiles of *TabsGST* Genes” section. The experimental design of real-time quantitative PCR included four biological replicates per treatment group and three technical replicates per sample. The RT-qPCR amplification conditions remained identical to those specified for the developmental expression analysis.

### Statistical Analysis

The data from the laboratory bioassays were analyzed using IBM SPSS Statistics v23.0. Probit analysis was employed to fit the toxicity regression lines. For second-instar larvae of *T. absoluta* exposed to the two insecticides, the median lethal concentrations LC_50_ and their 95% confidence intervals (CIs), regression slopes ± standard errors (SE), chi-square values, and degrees of freedom (*df*) were calculated.

Relative mRNA expression levels were calculated using the 2^-ΔΔCt^ method. To evaluate expression differences across developmental stages, data were analyzed using one‑way analysis of variance (ANOVA) followed by Duncan’s multiple range test (DMRT) using IBM SPSS Statistics v23.0. For comparisons between different insecticide treatments, an independent ­samples *t*‑test was employed. All results are presented as means ± standard errors (SE) of independent biological replicates, and statistical significance was established at a threshold of *P *< 0.05.

## Results

### Genome-Wide Identification and Characterization of *T. absoluta* GSTs

Genome-wide analysis and experimental validation (RT-qPCR and transcriptome-based) identified 23 GST genes in *T. absoluta*, categorized into 20 cytosolic and three microsomal isoforms (Table 3). The amino acid sequences of these TabsGSTs range from 147 to 289 aa in length. Bioinformatics characterization revealed that the encoded proteins exhibit predicted isoelectric points (pI) of 4.84 to 9.92 and molecular weights of 16.37 to 30.40 kDa, respectively.

**Table 3.** Characteristics of the 23 GST genes identified in *Tuta absoluta*

Type	Group	Gene name	Gene ID	Length (aa)	pI	M_W_ (kDa)
Cytosolic GSTs	Delta	*TabsGSTd1*	Tabs000152.1	215	6.90	24.11
		*TabsGSTd2*	Tabs009016.1	218	4.84	24.56
	Epsilon	*TabsGSTe1*	Tabs001420.1	217	7.62	25.17
		*TabsGSTe2*	Tabs007326.1	225	8.53	25.16
		*TabsGSTe3*	Tabs007826.1	223	5.88	24.85
		*TabsGSTe4*	Tabs009288.1	245	9.21	28.45
		*TabsGSTe5*	Tabs010796.1	250	9.14	28.84
		*TabsGSTe6*	Tabs015693.1	252	9.01	28.72
		*TabsGSTe7*	Tabs018311.1	191	5.12	21.72
		*TabsGSTe8*	Tabs019985.1	229	8.46	25.57
	Theta	*TabsGSTt1*	Tabs000292.1	221	9.14	25.94
	Sigma	*TabsGSTs1*	Tabs013460.1	203	8.47	23.88
		*TabsGSTs2*	Tabs021167.1	204	6.75	23.38
	Zeta	*TabsGSTz1*	Tabs000190.1	214	8.84	24.45
		*TabsGSTz2*	Tabs008759.1	222	7.07	25.56
	Omega	*TabsGSTo1*	Tabs003803.1	241	7.01	28.69
		*TabsGSTo2*	Tabs003889.1	254	6.25	29.31
		*TabsGSTo3*	Tabs017552.1	289	7.63	33.40
	Unclassified	*TabsGSTu1*	Tabs011472.1	231	6.23	26.38
		*TabsGSTu2*	Tabs016947.1	217	6.73	24.42
Microsomal GSTs		*TabsGSTm1*	Tabs000541.1	151	9.63	16.96
		*TabsGSTm2*	Tabs007841.1	148	9.92	16.37
		*TabsGSTm3*	Tabs020974.1	147	9.78	16.51

**Fig. 1.**
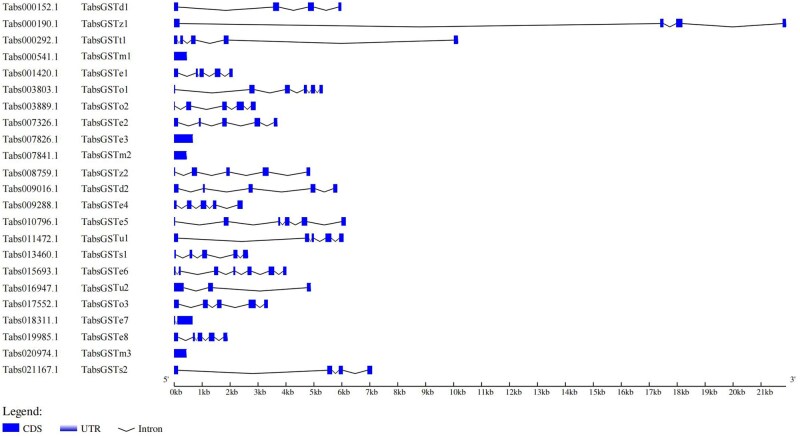
Gene structure of *TabsGST* gene family. Blue boxes represent exons, while black lines represent introns.

The genomic architecture of *TabsGSTs* exhibits considerable diversity in exon-intron organization (Fig. 1). Four genes (*TabsGSTm1/m2/m3* and *TabsGSTe3*) are composed of a single exon, whereas *TabsGSTe7* and *TabsGSTu2* contain two and three exons, respectively. A four-exon structure was identified in *TabsGSTd1*, *TabsGSTz1*, and *TabsGSTs2*. The largest group, comprising 11 genes (*TabsGSTt1*, *TabsGSTe1*, *TabsGSTo2*, *TabsGSTe2*, *TabsGSTz2*, *TabsGSTd2*, *TabsGSTe4*, *TabsGSTu1*, *TabsGSTs1*, *TabsGSTo3* and *TabsGSTe8*), contains four exons. More complex arrangements were observed in *TabsGSTo1* and *TabsGSTe5* (six exons), with *TabsGSTe6* possessing the highest number of exons (seven), representing the most complex gene structure within this family.

### Chromosomal Mapping of *TabsGST* Genes

**Fig. 2.**
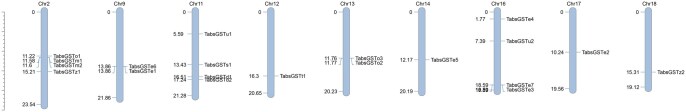
Chromosomal distribution of *TabsGST* genes in the *T. absoluta* genome.

Chromosomal mapping localized 20 *TabsGST* genes across nine chromosomes within the *T. absoluta* genome (Fig. 2). The genes exhibited a non-random distribution pattern: while Chr12, 14, 17, and 18 each possessed a single *TabsGST* locus, Chr9 and 13 each contained two loci. Notably, Chr2, 11, and 16 displayed the highest gene density, with each harboring four *TabsGST* members. This uneven distribution highlights potential gene duplication events on specific chromosomes.

### Evolutionary Relationships and Classification

**Fig. 3.**
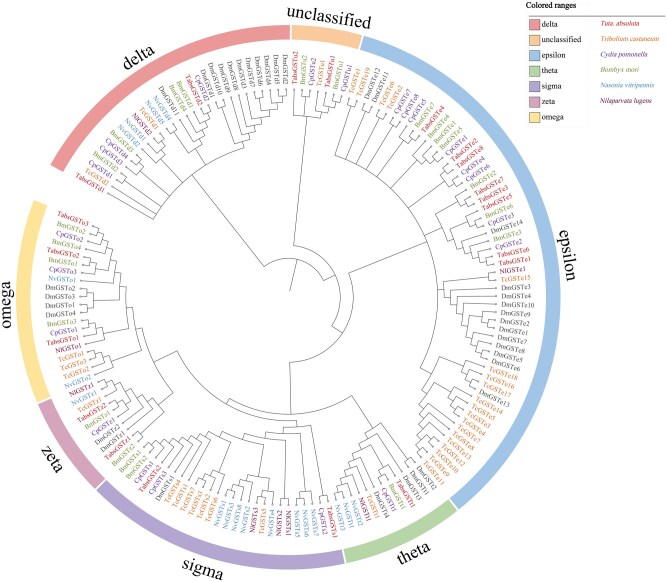
Phylogenetic relationships of GSTs from *T. absoluta* and representative insects.

Phylogenetic reconstruction assigned the 20 cytosolic *TabsGSTs* to seven canonical subfamilies, alongside GSTs from six representative insect taxa (Fig. 3). The *T. absoluta* GST repertoire includes the following subfamilies: Delta (two), Epsilon (eight), Sigma (two), Zeta (two), Omega (three), Theta (one), and two unclassified members. Notably, the Epsilon class exhibited the highest degree of expansion, indicating that lineage-specific gene duplication events likely shaped the diversity of this subfamily in *T. absoluta*.

### Spatiotemporal Expression Analysis Across Life Stages

**Fig. 4.**
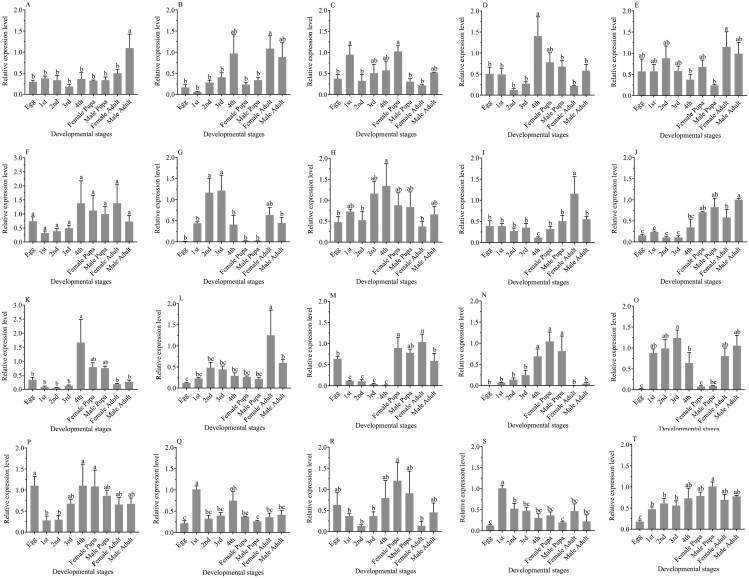
Transcriptional profiles of 20 *TabsGST* genes across various life stages of *T. absoluta*. Sub-panels (A–T) correspond to individual *TabsGST* members as listed: (A) *TabsGSTd1*, (B) *TabsGSTz1*, (C) *TabsGSTt1*, (D) *TabsGSTe1*, (E) *TabsGSTo1*, (F) *TabsGSTo2*, (G) *TabsGSTe2*, (H) *TabsGSTe3*, (I) *TabsGSTz2*, (J) *TabsGSTd2*, (K) *TabsGSTe4*, (L) *TabsGSTe5*, (M) *TabsGSTu1*, (N) *TabsGSTs1*, (O) *TabsGSTe6*, (P) *TabsGSTu2*, (Q) *TabsGSTo3*, (R) *TabsGSTe7*, (S) *TabsGSTe8*, and (T) *TabsGSTs2*. Egg: egg stage; 1st–4th: first to fourth instar larvae.

RT-qPCR analysis demonstrated that *TabsGST* genes exhibit distinct stage-specific expression profiles in *T. absoluta* (Fig. 4). The egg stage was characterized by the predominant expression of *TabsGSTo1*, *TabsGSTu2*, and *TabsGSTe7*. In contrast, 13 genes (*TabsGSTz1*, *TabsGSTt1*, *TabsGSTe1*, *TabsGSTo1*, *TabsGSTe2*, *TabsGSTe3*, *TabsGSTe4*, *TabsGSTe6*, *TabsGSTu2*, *TabsGSTo3*, *TabsGSTe7*, *TabsGSTe8*, and *TabsGSTs2*) exhibited significantly higher expression levels across all larval instars than the other genes, while 10 members (*TabsGSTt1*, *TabsGSTe1*, *TabsGSTo1*, *TabsGSTe3*, *TabsGSTd2*, *TabsGSTe4*, *TabsGSTu1*, *TabsGSTu2*, *TabsGSTe7*, and *TabsGSTs2*) were highly expressed in the pupal stage. The most extensive expression profile was observed in the adult stage, where 15 *TabsGST* members (including *TabsGSTd1*, *TabsGSTz1*, *TabsGSTt1*, *TabsGSTo1*, *TabsGSTe2*, *TabsGSTe3*, *TabsGSTz2*, *TabsGSTd2*, *TabsGSTe5*, *TabsGSTu1*, *TabsGSTs1*, *TabsGSTe6*, *TabsGSTu2*, *TabsGSTe7*, and *TabsGSTs2*) exhibited significantly higher expression levels than the other genes. These findings highlight the dynamic transcriptional regulation of GSTs throughout the insect’s life cycle, with the adult and larval stages possessing the most diverse repertoire of active GST genes.

### Results of Laboratory Toxicity Bioassays

As detailed in Table 4, laboratory bioassays indicated that the Haidian population of *T. absoluta* second-instar larvae was susceptible to both chlorantraniliprole and spinetoram. The LC_50_ values were 0.216 mg/L (95% CI: 0.182 ∼ 0.257 mg/L; *df *= 16; *P *= 1.000) for chlorantraniliprole and 0.339 mg/L (95% CI: 0.283 ∼ 0.409 mg/L; *df *= 16; *P *= 0.998) for spinetoram. These toxicological parameters established the critical baseline required for subsequent sublethal exposure experiments aimed at screening key detoxification genes in *T. absoluta*.

**Table 4.** Toxicity bioassays of two agents against *Tuta absoluta*

Insecticide	Slope ± SE	LC_50_ (mg/L)	95% confidence interval (mg/L)	*X* ^2^	*df*	*P*
Chlorantraniliprole	2.05 ± 0.185	0.216	0.182 ∼ 0.257	3.158	16	1
Spinetoram	2.01 ± 0.18	0.339	0.283 ∼ 0.409	4.552	16	0.998

### Transcriptional Response to Chlorantraniliprole Exposure

**Fig. 5.**
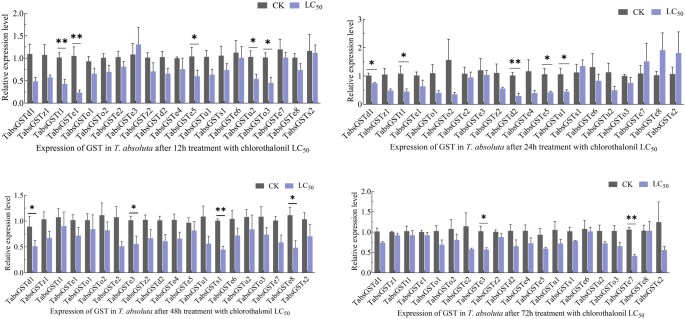
Transcriptional response of *TabsGSTs* in *T. absoluta* second-instar larvae under chlorantraniliprole LC_50_ treatment. Data are presented for 12, 24, 48, and 72 h post-exposure. Asterisks denote statistical significance compared to the control (*P < 0.05, **P < 0.01; independent samples *t*-test).

RT-qPCR analysis revealed significant reductions in the expression levels of *TabsGSTe5* (at 12 and 24 h), *TabsGSTd1* (at 24 and 48 h), and *TabsGSTe3* (at 48 and 72 h) (Fig. 5). These temporal expression profiles suggest a selective inhibitory effect of LC_50_ chlorantraniliprole exposure on specific members of the *TabsGST* gene family over time.

### Transcriptional Response to Spinetoram Exposure

**Fig. 6.**
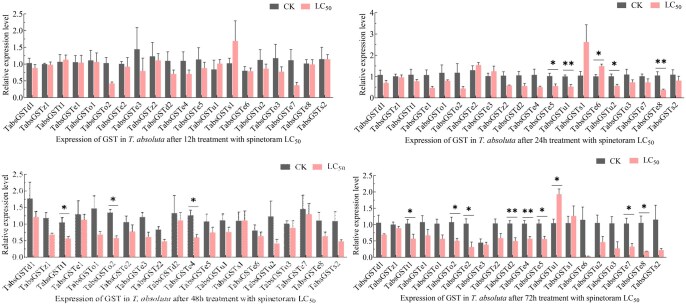
Transcriptional response of *TabsGSTs* in *T. absoluta* second-instar larvae under spinetoram LC_50_ treatment. Data are shown for 12, 24, 48, and 72 h post-exposure. Asterisks denote statistical significance compared to the control group (* *P *< 0.05, ** *P *< 0.01; independent samples *t*-test).

As shown in Fig. 6, exposure to spinetoram at the LC_50_ generally suppressed the expression of most *TabsGST* members in *T. absoluta* larvae, with the exception of a few induced genes. Specifically, *TabsGSTe5* (at 24 and 72 h), along with *TabsGSTe4*, *TabsGSTt1*, and *TabsGSTo2* (at 48 and 72 h), were significantly downregulated. Notably, *TabsGSTe4* exhibited extremely significant downregulation at 72 h. Interestingly, *TabsGSTu1* was significantly downregulated at 24 h but shifted to significant upregulation by 72 h, whereas *TabsGSTe6* was significantly upregulated at 24 h. This differential response indicates that while the majority of *TabsGSTs* are suppressed under LC_50_ spinetoram stress, specific members such as *TabsGSTe6* and *TabsGSTu1* represent key candidate genes transcriptionally responsive to this insecticide.

## Discussion

The persistent reliance on chemical pesticides has been a primary driver for the induction or inhibition of detoxification enzyme activities, ultimately fostering insecticide resistance in *T. absoluta*. The diversification of detoxification gene families is instrumental in insect adaptation to ecological niches and their survival under xenobiotic pressure (Strode et al. 2008). Among these systems, glutathione *S*-transferases (GSTs) play pivotal roles in metabolic detoxification, molecular transformation, and antioxidant defense (Enayati et al. 2005, Li et al. 2007). In this study, we systematically characterized the cytosolic GST gene family in *T. absoluta*. Our genome-wide analysis identified a repertoire of 23 GST genes, a count that is consistent with other Lepidopterans, such as 25 GSTs in *C. pomonella* (Hu et al. 2022), 31 GSTs in *S. frugiperda* (Aioub et al. 2023), 19 GSTs in *P. xylostella* (Chen and Zhang 2015), and 23 GSTs in *C. medinalis* (Liu et al. 2015). Notably, the Epsilon subfamily emerged as the most abundant subclass in *T. absoluta*, suggesting its dominant role in xenobiotic metabolism and oxidative stress responses.

Structural analysis revealed that *TabsGST* genes possess varied exon-intron organizations, with exon numbers ranging from one to seven. While the five-exon structure predominated, *TabsGSTe6* exhibited the most complex genomic architecture. Such structural diversity provides a molecular entry point for understanding adaptive evolution and designing precision pest control strategies (Gompert et al. 2025). Furthermore, we identified four tandemly arranged gene clusters: *TabsGSTm1/m2* (Chr 2), *TabsGSTe1/e6* (Chr 9), *TabsGSTo2/o3* (Chr 13), and *TabsGSTe3/e7* (Chr 16). These tandem gene clusters thus represent possible gene duplication events occurring on the chromosomes. Notably, due to incomplete scaffold assembly, three genes (*TabsGSTe8*, *TabsGSTs2*, and *TabsGSTm3*) have not yet been assigned to specific chromosomal positions.

Our results identified 13 *TabsGST* genes with high expression during the larval stage. As the primary feeding phase, larvae are frequently exposed to dietary toxins and plant secondary metabolites; thus, these GSTs likely facilitate the neutralization of ingested xenobiotics (You et al. 2015). It is noteworthy that at the adult stage, 15 genes exhibited significantly higher expression levels than the remaining *TabsGST* members. Such stage-specific differential expression is a widespread phenomenon in insects (You et al. 2015, Sun et al. 2020), likely reflecting the physiological demands of reproduction or hormonal regulation, though the precise mechanisms in *T. absoluta* require further investigation.

In response to the current *T. absoluta* outbreaks, chemical intervention, particularly the use of chlorantraniliprole and spinetoram, remains indispensable. Despite their divergent modes of action, their potential metabolic cross-talk via shared detoxification enzymes warrants careful consideration in rotational programs. As key Phase II enzymes, GSTs neutralize electrophilic xenobiotics through GSH conjugation (Xu et al. 2005). Interestingly, our results revealed that upon exposure to the LC_50_ of chlorantraniliprole, the expression of *TabsGST* genes in *T. absoluta* larvae was either downregulation or remained stable. This non-inductive response suggests that initial exposure might fail to trigger the larvae’s metabolic defenses or could actively suppress the transcription of specific *TabsGST* members (Jin et al. 2018), highlighting the complex physiological interactions between this insecticide and the insect’s enzymatic repertoire.

Exposure to the LC_50_ of spinetoram elicited highly variable responses across the gene family. At this concentration, while most members were either suppressed or remained stable, *TabsGSTe6* and *TabsGSTu1* were selectively induced at 24 h. This isoform-specific regulation may explain why only a restricted number of *TabsGST* genes are potentially recruited under spinetoram stress, whereas the remainder are transcriptionally suppressed (Daniel 1993). These findings highlight the sophisticated and diverse metabolic strategies insects utilized to navigate xenobiotic pressure. However, transcriptional upregulation data alone are insufficient to distinguish between adaptive and non-specific responses, nor can they definitively infer causal gene functions. Consequently, direct functional validation must be achieved through targeted approaches, such as mutant analysis, *in vitro* enzyme activity assays, metabolite identification, RNA interference (RNAi), or CRISPR-based gene editing (Pardo et al. 2025).

In summary, because only surviving individuals are sampled following LC_50_ treatment, our expression data inherently reflect the characteristics of a survivor subpopulation rather than the net effect of the insecticide on the entire population. Unlike studies utilizing lower sublethal concentrations (eg LC_10_–LC_30_) for physiological analyses—which avoid the selection bias induced by high mortality—future investigations aimed at distinguishing between these two possibilities should employ minimal sublethal concentrations and conduct expression analyses on all exposed individuals, rather than solely on survivors.

This study provides a systematic genome-wide characterization of the GST gene family in *T. absoluta*, uncovering its complex genomic architecture and evolutionary patterns. Our analysis of developmental and insecticide-stressed expression profiles highlights the critical involvement of GSTs in the insect’s adaptive responses to chemical stress. While these findings underscore the putative role of GSTs in the detoxification of chlorantraniliprole and spinetoram, further research into the synergistic effects of multiple detoxification pathways is warranted. Furthermore, because exposure to different insecticide concentrations may activate distinct detoxification genes, future dose-response transcriptional analyses are necessary (Martín-Blázquez et al. 2023). These insights offer crucial molecular targets and a robust theoretical framework for developing sustainable resistance monitoring programs and optimized chemical control strategies against *T. absoluta*.
